# Development and validation of a clinical prognostic model for BRAF V600E-mutated colorectal cancer patients based on pathological stage, microsatellite status, and primary tumor site

**DOI:** 10.3389/fonc.2024.1461237

**Published:** 2024-10-11

**Authors:** Kai Ou, Xiu Liu, Xiaoting Ma, Lin Yang

**Affiliations:** Medical Oncology, National Cancer Center/National Clinical Research Center for Cancer/Cancer Hospital, Chinese Academy of Medical Sciences and Peking Union Medical College, Beijing, China

**Keywords:** colorectal cancer, BRAF V600E mutated, clinical prognostic model, pathological stage, microsatellite status, primary tumor site

## Abstract

**Objective:**

To develop and validate a prognostic model for patients with BRAF V600E-mutated colorectal cancer.

**Methods:**

The clinical and pathological information of 206 patients with BRAF V600E-mutated colorectal cancer diagnosed in Cancer Hospital, Chinese Academy of Medical Sciences, and Peking Union Medical College from 2014 to 2021 was retrospectively collected. Least absolute shrinkage and selection operator (LASSO) regression, Cox regression, and nomograms were used to develop clinical prognostic models. The differentiation was measured using C-statistic, and the predicted variability was evaluated using the calibration curve. The prognostic model was externally validated using validation set data from 164 patients pooled from five studies.

**Results:**

Our clinical prognostic model included three variables: pathological stage, microsatellite status, and primary tumor site. In internal validation, the model had a concordant index of 0.785 (95% CI [0.732–0.839]) and a concordant index of 0.754 (95% CI [0.698–0.810]) using pathological staging. External validation confirmed the robustness of the model with a consistency index of 0.670 (95% CI [0.617–0.724]) and a consistency index of 0.584 (95% CI [0.546–0.622]) using pathological staging. Likelihood ratio test results show that our model is better (internal validation, p = 5.141e−03; external validation, p = 2.728e−05). The calibration graph drawn based on the prediction and the actual situation is close to the 45° diagonal.

**Conclusion:**

By adding microsatellite status and primary tumor site on the basis of pathological stage, we improved the discriminability and prediction accuracy of the model and successfully established a prognosis model for patients with BRAF V600E mutation of colorectal cancer.

## 1 Introduction

It was estimated that there were more than 1.9 million new cases of colorectal cancer (CRC) worldwide in 2020, and the number of deaths was approximately 935,000, accounting for approximately 10% of the total new cases and deaths of cancer in 2020. Data showed that the overall incidence of CRC ranks third in the world, and the mortality rate ranks second (1). Less than 10% of primary CRC and 5.1%–8.2% of metastatic CRC (mCRC) patients have mutations in the v-raf murine sarcoma viral oncogene homolog B (BRAF) gene (2–5). Among them, BRAF V600E mutation is the most common, accounting for approximately 90% (6).

The prognosis of mCRC patients with BRAF V600E mutation is poor, with the median overall survival (OS) reported in the literature ranging from 10 to 20 months (7). However, approximately 10%–20% of patients survived for more than 24 months after diagnosis of BRAF V600E-mutated mCRC (8–10). These results suggest that CRC patients with BRAF V600E mutation may be a heterogeneous group with different prognoses, and the clinical and molecular pathological factors related to their prognosis need further study.

The current methods for detecting BRAF V600E mutation include “next-generation” sequencing technology (NGS), polymerase chain reaction (PCR), and immunohistochemistry (IHC) (11). Since 2013, the Cancer Hospital of the Chinese Academy of Medical Sciences has adopted VENTANA anti-BRAF V600E (VE1) Mouse Monoclonal Primary Antibody as one of the standard reagents for the IHC method of detecting BRAF V600E mutations. This reagent was approved by the Food and Drug Administration (FDA) (12) in 2017 and by the National Medical Products Administration (NMPA) (13) in 2015 for related testing. The regulatory agency has clear regulations on the testing process and results. The data analysis of this research institution (14) and many other research institutions (15, 16) also showed that the sensitivity and specificity of this detection method reached more than 98%, the operation difficulty was small, and the detection cost was low (17).

In recent years, clinical prediction models including nomograms have become increasingly popular with oncologists because they provide more personalized estimates of recurrence and survival compared with traditional TNM staging (9). The Center for Precision Medicine of the American Joint Committee on Cancer (AJCC) endorsed the clinical prediction model and issued technical guidelines for the development of nomograms (18). The TRIPOD (Transparent reporting of a multivariable prediction model for individual prognosis or diagnosis) statement and interpretation formed by Oxford University in 2011 (https://www.tripod-statement.org/) also made more standardized requirements for the construction of clinical prediction models (19, 20).

The purpose of this study was to develop and validate a prognostic model for BRAF V600E-mutated CRC patients based on the clinical and pathological information of BRAF V600E-mutated CRC patients in order to distinguish subgroups with different prognoses at an early stage and adopt targeted treatment strategies.

## 2 Patients and methods

### 2.1 Patient cohort

The clinical and pathological information of 220 patients with BRAF V600E-mutated CRC diagnosed at the Cancer Hospital of the Chinese Academy of Medical Sciences from 2014 to 2021 was collected retrospectively. The inclusion criteria were as follows: 1) over 18 years old, 2) tumor resection and histopathologically confirmed CRC, and 3) tumor tissue confirmed to have BRAF V600E mutation by IHC. The exclusion criterion was as follows: 1) second primary malignant tumors in other organs or systems. The validation set used BRAF V600E-mutated CRC patient data from five studies (four of which were from the cBioPortal database, https://www.cbioportal.org/) (21–25). The study was approved by the institutional ethics committee.

### 2.2 Variable

The training set included age, gender, primary tumor site, pathological stage, Eastern Cooperative Oncology Group Performance Status (ECOG PS) score, smoking status, drinking status, whether radical surgery was performed, microsatellite status, histopathological grade, preoperative radiotherapy, preoperative chemotherapy, postoperative radiotherapy, postoperative chemotherapy, blood carcinoembryonic antigen (CEA), carbohydrate antigen 19-9 (CA19-9), albumin (ALB), lactate dehydrogenase (LDH), alkaline phosphatase (ALP), hemoglobin, white blood cells, neutrophils, lymphocytes and the ratio of neutrophils to lymphocytes at the time of diagnosis; there were 24 variables. BRAF V600E mutations were detected by immunohistochemistry (14). Microsatellite instability was detected by immunohistochemistry (26, 27). Microsatellite instability causes abnormal accumulation of short repetitive sequences (microsatellites) throughout the genome. Immunohistochemical staining for mismatch repair proteins identifies >94% of microsatellite instability (MSI) tumors and has become the standard for pathology reporting (26, 27).

### 2.3 Study endpoint

The study endpoint was OS, defined as the interval between diagnosis of CRC and death from any cause or last follow-up.

### 2.4 Development and validation of clinical prediction models

The least absolute shrinkage and selection operator (LASSO) method is suitable for regression on high-dimensional data and selecting the most useful predictive features from the original dataset (28). Threefold cross-validation was used in the LASSO model to select the smallest λ-value screening variable. The selected predictive features were assessed for the association of relevant clinical and pathological variables with OS using Cox proportional hazards regression models. Proportional hazards assumptions were validated by temporal correlation tests and residual plot inspections. The Akaike information criterion (AIC) was used to select variables backward stepwise for the identification of a multivariate Cox proportional hazards regression model. The 95% CI of the hazard ratio (HR) was calculated. Nomograms of selected variables were constructed using R (version 4.1.0; http://www.r-project.org). All statistical tests were two-tailed with a significance level of 0.05. To assign scores to features in the nomogram, regression coefficients were applied to each feature to define linear predictors.

Internal validation of the multivariate Cox proportional hazards regression model was performed using the concordance index (C index) and calibration curve, and the difference in the likelihood ratio test and C index between the model and pathological stage modeling alone was compared. Similarly, the BRAF V600E mutation CRC patient data of five studies (four of which came from the cBioPortal database) were used for external validation; that is, the C index of the validation set was calculated, the calibration curve of the validation set was drawn, likelihood ratio test was performed, and the C index difference between the model and pathological stage modeling in the validation set was compared.

The median of the nomogram prediction scores of the training set was used to distinguish the high- and low-risk groups, and the Kaplan–Meier curves of the training set and the validation set were drawn.

## 3 Results

### 3.1 Enrollment process

**Figure 1:**
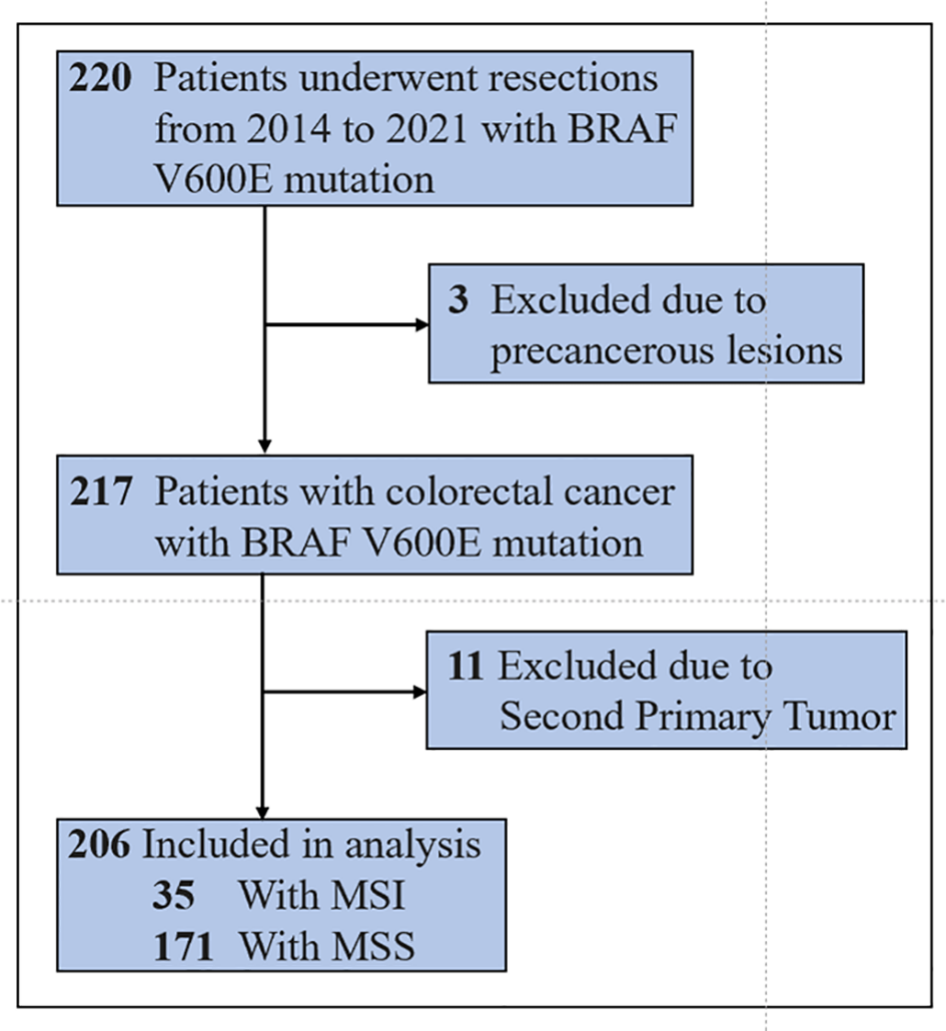
Flowchart.

A total of 220 patients with BRAF V600E-mutated colorectal cancer diagnosed at the Cancer Hospital of the Chinese Academy of Medical Sciences from 2014 to 2021 were included. Among them, three patients were only pathologically confirmed as having precancerous lesions, and 11 patients had second primary tumors. Finally, 206 patients were included in the training set. By the last follow-up on August 15, 2022, 55 patients had events (death), 22 were censored, and 129 survived (see **Figure 1**). The median overall survival of all patients in the training set was not reached (NR). The median follow-up time of surviving patients was 71.8 months.

### 3.2 Patient characteristics

The baseline characteristics of the training set and validation set are listed in **Table 1**, and the data of these patients were used to develop the clinical prognosis model.

Table 1AThe baseline characteristics of the training set.

**Table:** 

	No. of patients (%)		
Characteristic (at diagnosis)	Full cohort	MSI	MSS
	(N = 206)	(n = 35)	(n = 171)
Age, years^a^	61 (21–84)	65 (29–84)	60 (21–82)
Sex			
Male	120 (58)	13 (37)	107 (63)
Female	86 (42)	22 (63)	64 (37)
Site			
Right	66 (32)	21 (60)	45 (26)
Left	69 (33)	9 (26)	60 (35)
Rectum	71 (35)	5 (14)	66 (39)
Stage			
I	15 (7)	2 (6)	13 (8)
II	66 (32)	19 (54)	47 (27)
III	91 (44)	12 (34)	79 (46)
IV	34 (17)	2 (6)	32 (19)
T category			
0	2 (1)	0 (0)	2 (1)
1	4 (2)	0 (0)	4 (2)
2	22 (11)	2 (6)	20 (12)
3	86 (42)	19 (54)	67 (39)
4	92 (44)	14 (40)	78 (46)
N category			
0	84 (41)	22 (63)	62 (36)
1	68 (33)	11 (31)	57 (33)
2	54 (26)	2 (6)	52 (31)
M category			
0	172 (83)	33 (94)	139 (81)
1	34 (17)	2 (6)	32 (19)
ECOG PS			
0	184 (89)	31 (89)	153 (89)
1	17 (8)	4 (11)	13 (8)
≥2	5 (3)	0 (0)	5 (3)
Smoking^b^			
<10	132 (64)	24 (69)	108 (63)
≥10	74 (36)	11 (31)	63 (37)
Alcohol drinking			
Moderate	174 (84)	30 (86)	144 (84)
Excessive	32 (16)	5 (14)	27 (16)
Histologic grade (differentiation)			
Well or moderate	96 (47)	11 (31)	85 (50)
Poor	110 (53)	24 (69)	86 (50)
Operation			
Radical	180 (87)	33 (94)	147 (86)
Palliative	26 (13)	2 (6)	24 (14)
Preoperative radiotherapy			
Yes	14 (7)	5 (14)	9 (5)
No	192 (93)	30 (86)	162 (95)
Preoperative chemotherapy			
Yes	37 (18)	8 (23)	29 (17)
No	169 (82)	27 (77)	142 (83)
Postoperative radiotherapy			
Yes	21 (10)	1 (3)	20 (12)
No	185 (90)	34 (97)	151 (88)
Postoperative chemotherapy			
Yes	117 (57)	16 (46)	101 (59)
No	89 (43)	19 (54)	70 (41)
CEA^c^			
Normal	122 (59)	22 (63)	100 (58)
Elevated	84 (41)	13 (37)	71 (42)
CA19-9^c^			
Normal	165 (80)	24 (69)	141 (82)
Elevated	41 (20)	11 (31)	30 (18)
ALB^c^			
Decreased	8 (4)	5 (14)	3 (2)
Normal	197 (95)	29 (83)	168 (98)
Elevated	1 (1)	1 (3)	0 (0)
LDH^c^			
Decreased	35 (17)	8 (23)	27 (16)
Normal	164 (80)	25 (71)	139 (81)
Elevated	7 (3)	2 (6)	5 (3)
ALP^c^			
Decreased	3 (1)	0 (0)	3 (2)
Normal	201 (98)	35 (100)	166 (97)
Elevated	2 (1)	0 (0)	2 (1)
Hb			
Decreased	69 (34)	20 (57)	49 (28)
Normal	128 (62)	14 (40)	114 (67)
Elevated	9 (4)	1 (3)	8 (5)
WBC			
Decreased	8 (4)	0 (0)	8 (5)
Normal	189 (92)	29 (83)	160 (93)
Elevated	9 (4)	6 (17)	3 (2)
Neutrophilic granulocyte (NE)			
Decreased	11 (5)	0 (0)	11 (7)
Normal	187 (91)	31 (89)	156 (91)
Elevated	8 (4)	4 (11)	4 (2)
Lymphocyte (LYM)			
Decreased	6 (3)	0 (0)	6 (4)
Normal	199 (96)	34 (97)	165 (96)
Elevated	1 (1)	1 (3)	0 (0)
NLR			
<2	79 (38)	6 (17)	73 (43)
≥2	127 (62)	29 (83)	98 (57)

MSI, microsatellite instability; MSS, microsatellite stability; ECOG PS, Eastern Cooperative Oncology Group Performance Status; CEA, carcinoembryonic antigen; CA19-9, carbohydrate antigen 19-9; ALB, albumin; LDH, lactate dehydrogenase; ALP, alkaline phosphatase; Hb, hemoglobin; WBC, white blood cell; NLR, neutrophil-to-lymphocyte ratio; NE, neutrophilic granulocyte; LYM, lymphocyte. ^a^, Age is measured in years; ^b^, The unit of 'smoking' is pack-year; ^c^, The normal range of CEA is 0.0-5.0 ng/ml, CA19-9 is 0.0-3.3 ng/ml, ALB is 40.0-55.5 g/L, LDH is 120.0-250.0 U/L, ALP is 50.0-135.0 U/L.

**Table 1B:** The baseline characteristics of the training set.

	No. of patients (%)		
Characteristic (at diagnosis)	Full cohort	MSI	MSS
	(N = 164)	(n = 65)	(n = 99)
Sex			
Male	65 (40)	22 (34)	43 (43)
Female	99 (60)	43 (66)	56 (57)
Site			
Left	22 (13)	3 (5)	19 (19)
Right/rectum	142 (87)	62 (95)	80 (81)
Stage			
I	3 (2)	2 (3)	1 (1)
II	11 (7)	11 (17)	0 (0)
III	18 (11)	13 (20)	5 (5)
IV	132 (80)	39 (60)	93 (94)

MSI, microsatellite instability; MSS, microsatellite stability.

### 3.3 Model establishment

**Figure 2:**
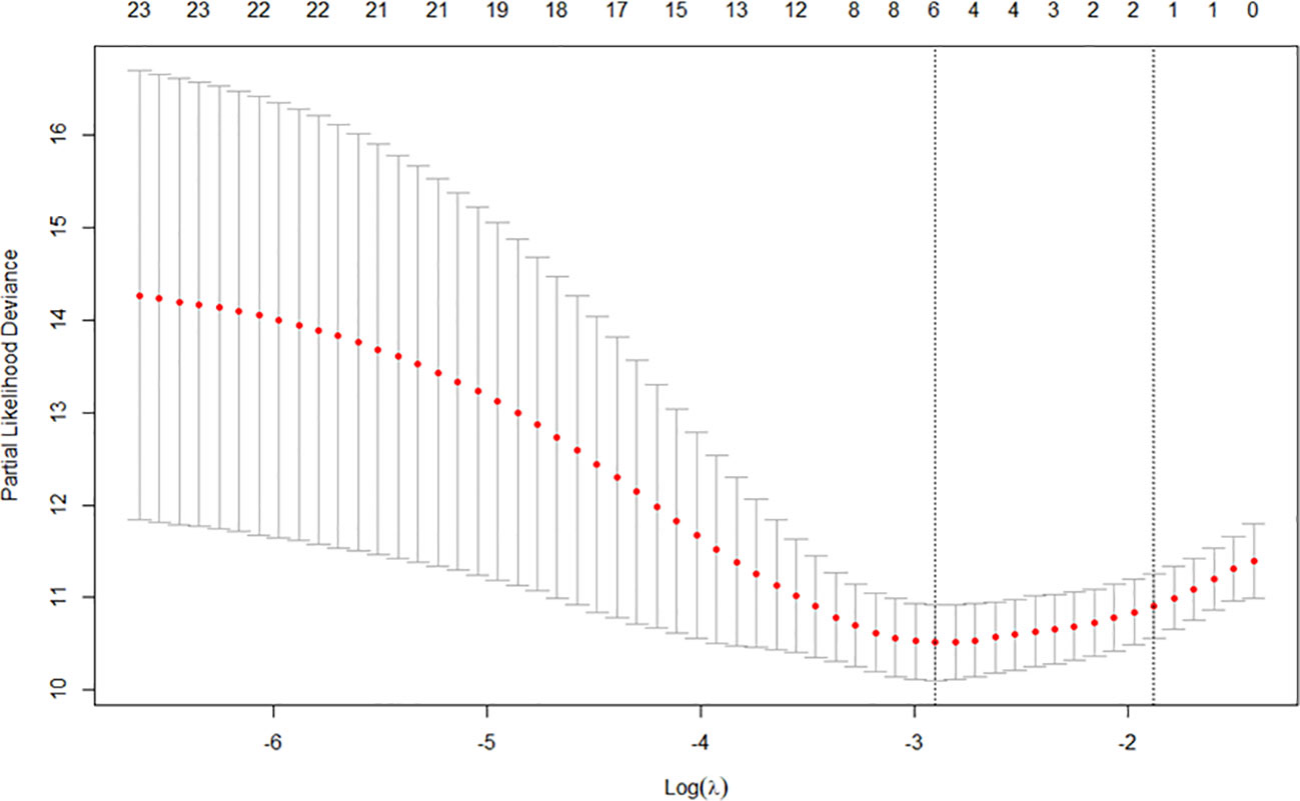
LASSO regression minimum λ value. LASSO, Least absolute shrinkage and selection operator.

Through LASSO regression, according to threefold cross-validation, the smallest λ value was selected to be 0.0548, and a total of six variables were screened out (see **Figure 2**), which were pathological stage, microsatellite status, primary tumor site, whether to undergo radical surgery, CEA, and CA19-9.

**Figure 3:**
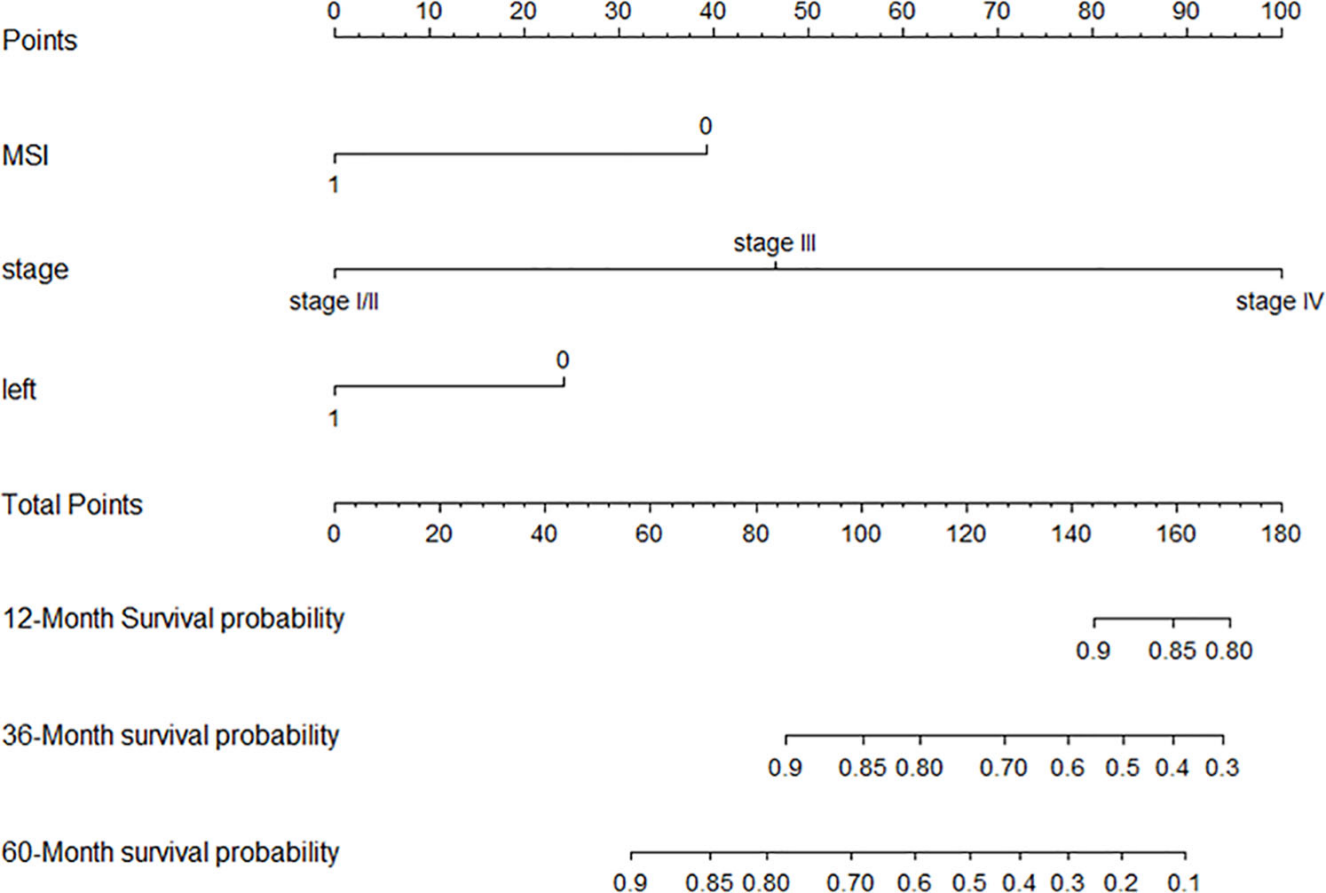
Nomogram.

Backward selection was then used to build multivariate models by adding statistically significant (p < 0.05) variables in the Cox model univariate analysis. The three variables included in the final multivariate model were tumor stage, primary tumor site, and microsatellite status (**Table 2**). The established nomogram is shown in **Figure 3**. **Supplementary Table 1** shows the specific scores of each variable in the nomogram and the calculation formula of survival probability at different time points.

**Table 2:** Cox regression analysis.

Variable	Univariable		Multivariable	
	HR (95% CI)	p-Value	HR (95% CI)	p-Value
Factors selected				
Stage				
IV	1 [Reference]	NA	1 [Reference]	NA
III	0.25 (0.14–0.44)	<0.001 **	0.37 (0.14–0.97)	0.044 *
I/II	0.06 (0.02–0.14)	<0.001 **	0.10 (0.03–0.32)	<0.001**
Microsatellite status				
MSS	1 [Reference]	NA	1 [Reference]	NA
MSI	0.22 (0.07–0.69)	0.01 **	0.30 (0.09–0.98)	0.047 *
Cancer of the left colon				
No	1 [Reference]	NA	1 [Reference]	NA
Yes	0.75 (0.42–1.34)	0.32	0.52 (0.28–0.96)	0.038 *
Radical surgery				
Yes	1 [Reference]	NA	1 [Reference]	NA
No	6.26 (3.52–11.14)	<0.001 **	2.10 (0.80–5.51)	0.131
CEA	0.999 (0.994–1.004)	0.725	0.997 (0.991–1.003)	0.348
CA19-9	1.001 (1.001–1.002)	<0.001 **	1.001 (1.000–1.001)	0.057

MSI, microsatellite instability; MSS, microsatellite stability; CEA, carcinoembryonic antigen; CA19-9, carbohydrate antigen 19-9. One asterisk (*): p-value less than 0.05. Two asterisks (**): p-value less than 0.01.

### 3.4 Model validation

**Figure 4:**
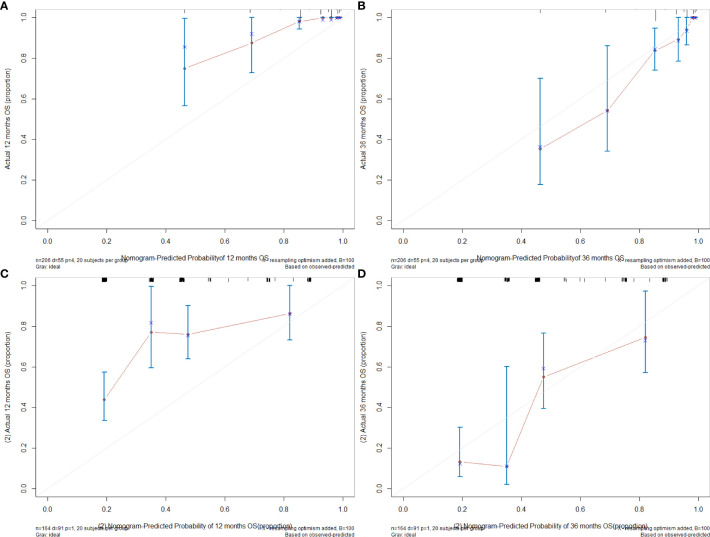
**(A)** The 12-month calibration graph of training set. **(B)** The 36-month calibration graph of training set. **(C)** The 12-month calibration graph of validation set. **(D)** The 36-month calibration graph of validation set.

For the training set, the C index of the nomogram model established using three variables (tumor location at diagnosis, pathological stage, and microsatellite status) was 0.785 (95% CI [0.732–0.839]), which was better than the C index of 0.754 (95% CI [0.698–0.810]) of the nomogram model established by pathological stages alone (the method was consistent). Similarly, the C index of the validation set using the nomogram model established by three variables was 0.670 (95% CI [0.617–0.724]), which was better than the C index of 0.584 (95% CI [0.546–0.622]) of the nomogram model established by pathological stages alone. Likelihood ratio test results show that our model was better (internal validation, p = 5.141e−03; external validation, p = 2.728e−05). The calibration graph drawn based on the prediction and the actual situation is close to the 45° diagonal (**Figure 4**).

### 3.5 Kaplan–Meier curve

**Figure 5:**
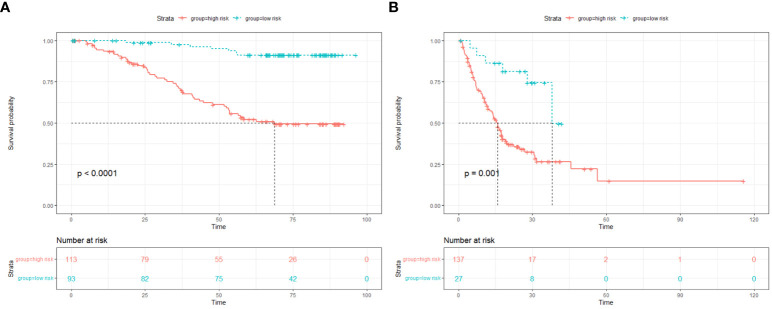
**(A)** K-M curve of the training set. **(B)** K-M curve of validation set. K-M, Kaplan–Meier.

The median of the nomogram prediction score of the training set was 82 to distinguish between high- and low-risk groups, and the Kaplan–Meier (K-M) curves of the training set and the verification set were drawn, as shown in **Figures 5A, B**.

## 4 Discussion

This study developed and validated a clinical prognostic model based on three factors, namely, pathological stage, microsatellite status, and primary tumor site, in patients with BRAF V600E-mutated CRC. This model successfully differentiated the prognosis of patients with BRAF V600E-mutated CRC.

The pathological stage is undoubtedly the most important factor affecting the prognosis of CRC patients with BRAF V600E mutation. Among the patients included in the training set of this study, patients with stage III accounted for the largest proportion, which is consistent with other studies (5, 29). Previously published analyses of the prognosis of BRAF V600E-mutated CRC mostly focused on the patient population with distant metastases (stage IV) (30–32). In this study, a clinical prognostic model was established for the prognostic factors of all BRAF V600E-mutated CRC patients in stages I–IV.

Most studies have reported that microsatellite status is associated with CRC prognosis. Among patients with stage II–III colorectal cancer, those with microsatellite instability have a better prognosis (33). The relationship between microsatellite status and prognosis in CRC patients with BRAF V600E mutation has rarely been reported. In this study, patients with microsatellite instability accounted for 17%, and the prognosis of patients with MSI was better than that of patients with microsatellite stability (MSS). A retrospective study showed that the proportion of microsatellite instability in BRAF mutant patients was much higher than that in BRAF wild-type CRC patients (54.8% vs. 11.5%) (34). A literature that included a small sample of patients reported that in stage IV CRC patients with BRAF V600E mutations who did not use immunotherapy, the proportion of MSI patients was higher among those who survived longer (32), and there is also a study showing that the prognosis of the above two groups is comparable (3).

Tumor location is one of the prognostic factors for BRAF V600E-mutated CRC patients, which is the most interesting finding of this study, in which only primary tumor location in the left-sided colon was a good prognosis factor, whereas location in the right-sided colon or rectum has poor prognosis factors. Right-sided cancer has a unique pathogenesis and poor overall prognosis (35–37). BRAF mutations most commonly occur in right-sided colon cancers. In this study, it was found that the prognosis of right-sided colon cancers with BRAF V600E mutations was worse than that of left-sided colon cancers. It has been reported in the literature that rectal cancer accounts for approximately 9.2%–27.7% (5, 29, 38) of BRAF V600E-mutated CRC. Rectal cancer accounted for 35% of our cohort. However, in many clinical trials including BRAF V600E-mutated CRC treatment (39), rectal cancer was often grouped with left-sided colon cancer for analysis. This study found that BRAF V600E-mutated rectal cancer had a worse prognosis than left-sided colon cancer. Rectal cancer with microsatellite instability was the least prevalent of all tumor locations in BRAF V600E-mutated CRC in this and other studies (38, 40). This may reflect the different clinical characteristics and prognosis of the BRAF V600E-mutated rectal cancer population from one aspect, and it is worthy of further exploration in the future.

The median diagnosis time of the patients included in the training set of this study was April 2016, so the vast majority of the patients who received medical treatment used the standard treatment of CRC, that is, oxaliplatin or irinotecan combined with fluorouracil drugs for chemotherapy. Neither preoperative nor postoperative chemotherapy was a statistically significant factor in LASSO regression and multivariate analysis. In the validation set, at least approximately 2/5 of the patients received targeted therapy based on BRAF inhibitors combined with epidermal growth factor receptor (EGFR) inhibitors, and a small number of patients with microsatellite instability received immunotherapy, but the 3-year survival probability of the validation set patients was still comparable to the predicted probability. Such results, on the one hand, illustrate the generalization of the model established in this study and, on the other hand, also indicate that changes in the current medical treatment plan have limited improvement in the survival of patients.

The advantage of this study is that on the basis of fully collecting the clinical and pathological information of patients including 24 variables in the training set, a clinical prognostic model in the form of a nomogram suitable for stage I-IV BRAF V600E-mutated CRC patients was established, and it has been verified in the verification set, which conforms to the TRIPOD specification. The limitations are the lack of data and analysis of the molecular biology of patients, the large differences in baseline characteristics of patients in the training set and validation set, including tumor stage, and the influence of selection bias inherent in observational retrospective studies.

This study suggests that for CRC patients with BRAF V600E mutation, the prognosis of patients can be stratified according to the patient’s stage, microsatellite status, and primary tumor site before treatment. Among patients with BRAF V600E mutation, patients with advanced stage, MSS, and primary tumors located in the right-sided colon or rectum have a poor prognosis, and more aggressive treatment strategies should be adopted.

## Data Availability

The original contributions presented in the study are included in the article/**Supplementary Material**. Further inquiries can be directed to the corresponding author.

## Supplementary material

Supplementary Table 1 The specific score of each variable and the calculation formula of survival probability at different time points.
